# Macular Hole Progression following Ocriplasmin Intravitreal Injection

**DOI:** 10.1155/2014/403461

**Published:** 2014-12-14

**Authors:** Edward Casswell, Guillermo Fernandez-Sanz, Danny Mitry, Sheila Luk, Rahila Zakir

**Affiliations:** ^1^Department of Ophthalmology, Western Eye Hospital, London NW1 5QH, UK; ^2^Moorfields Eye Hospital, London EC1V 2PD, UK

## Abstract

Ocriplasmin is a protease which has been approved for the treatment of symptomatic vitreomacular adhesion (VMA). A 63-year-old presented with blurred vision in the left eye and a best corrected visual acuity of 6/18. Optical coherence tomography revealed VMA with an underlying macular hole and she subsequently underwent a left intravitreal ocriplasmin injection. One week after the injection, VMA had been released but with enlargement of the macular hole and a drop in her BCVA to 6/60. This persisted at 1 month after the injection. It is important to warn patients that ocriplasmin may lead to an enlargement of their macular hole with resultant loss in visual acuity.

## 1. Introduction

Ocriplasmin is a recombinant protease which has been recently approved by FDA and NICE for the treatment of symptomatic vitreomacular adhesion (VMA) as an alternative to vitrectomy [1]. Studies have shown that it may facilitate nonsurgical closure of macular holes [2, 3]. We present a case of ocriplasmin leading to enlargement of a macular hole with resultant loss of visual acuity.

## 2. Case Report

**Figure 1:**
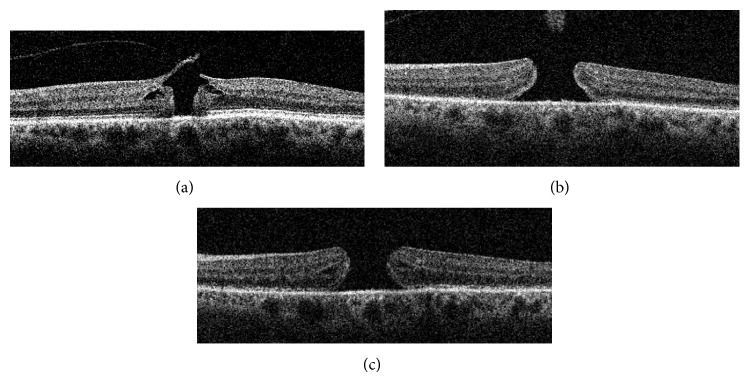
Spectral-domain optical coherence tomography (SD-OCT) 1 day prior (a), 14 days after (b), and 35 days after (c) intravitreal ocriplasmin injection. (a) Prior to ocriplasmin injection, there is vitreomacular adhesion (VMA), with an underlying macular hole (240 *µ*m diameter) involving the outer retinal layers. (b) 14 days after ocriplasmin injection, there is a resolution of VMA with enlargement of macular hole (540 *µ*m diameter), which is now at full thickness. (c) 35 days after ocriplasmin injections, the full thickness hole persists (556 *µ*m diameter).

A 63-year-old woman reported blurred vision in her left eye, with best corrected visual acuity (BCVA) dropping from 6/6 (0.0 logMAR) to 6/18 (0.48 logMAR). She had no past ophthalmic history and was phakic. Spectral-domain optical coherence tomography (SD-OCT) revealed VMA with an underlying macular hole with no associated epiretinal membrane (Figure 1(a)). One week following an uncomplicated intravitreal ocriplasmin injection (125 *µ*g in 0.10 mL), her BCVA was 6/60 (1.0 logMAR). Repeat SD-OCT revealed an enlarged full thickness macular hole (FTMH) with VMA release (Figure 1(b)). 35 days after injection, BCVA was 6/60 (0.92 logMAR) with a persistent enlarged FTMH (Figure 1(c)).

## 3. Comment

The MIVI-TRUST reported that versus placebo, ocriplasmin led to an increased frequency of macular hole closure (40.6% versus 10.1%) and VMA resolution [2]. Amongst adverse events, the study reported blurred vision in 8.6% of ocriplasmin patients but the majority of events were reported as transient. More recently, studies have suggested that ocriplasmin may lead to disruption of the ellipsoid layer, leading to transient visual loss and accumulation of subretinal fluid [3, 4]. Our case shows an increase in the size of a macular hole following ocriplasmin injection (Figures 1(a) and 1(c)), with associated loss in visual acuity.

Macular hole formation has previously been reported following intravitreal injections [5]. Indeed, MIVI-TRUST reported 8.6% macular hole formation in its placebo group and 5.2% in the ocriplasmin group [2]. In our case, although the intravitreal injection itself may have been a risk factor for formation of the macular hole, there is clear enlargement of the macular hole 1 week after ocriplasmin (Figures 1(a) and 1(b)). We postulate that, in this case, the movement of the vitreous body secondary to the intravitreal injection itself plus the cleavage effect of ocriplasmin on laminin and fibronectin led to a higher risk of macular hole enlargement. This may have been due to previously described ellipsoid layer disruption which has been linked to the development of subfoveal fluid following ocriplasmin injection [3]. Of note, in previous reports, ellipsoid layer abnormalities and subretinal fluid have resolved by 30 days [3, 4], whereas macular hole enlargement and decreased VA persisted in our case.

It is therefore important to advise patients when counselling them for ocriplasmin injections that it may lead to enlargement of their macular hole, with persistent worsening of their visual acuity.
